# Antioxidant Activities of *Aster glehni* Extracted with Different Solvents

**Published:** 2019-01

**Authors:** Sangwook SEO, Kisok KIM

**Affiliations:** 1. Gyeongsangbuk-do Government Public Institute of Health and Environment, Yeongcheon, Republic of Korea; 2. College of Pharmacy, Keimyung University, Daegu 42601, Republic of Korea

## Dear Editor-in-Chief

The *Aster glehni* F. Schmidt (family *Compositae*) is widely distributed on Ulleung Island in the Republic of Korea. It has been widely used as both a vegetable and a traditional herbal medicine to treat diabetes mellitus, hypercholesterolemia, insomnia, and cardiovascular disease (1). *A. glehni* has anticonvulsant, sedative, antioxidant, anti-inflammatory, and anti-adipogenic effects, as well as hypouricemic activity (2, 3). Natural products contain a wide variety of antioxidants, including phenolic acids and flavonoids (4). Most of the pharmacological activities of *A. glehni* are related to phenolic compounds (5). Since there have been no reports of quantitative phytochemical evaluations of *A. glehni*, the aim of this study was to examine the phenol and flavonoid content, and corresponding antioxidant activities, of *A. glehni* extracted with different solvents. Phenol and flavonoid contents were estimated using spectrophotometry according to the Korean National Institute of Food and Drug Safety Evaluation (NIFDS) Guidelines (6). 1, 1-diphenyl-2-picrylhydrazyl (DPPH) free radical scavenging activity was evaluated using the method described by Blois (7), and the method (8) was adopted to investigate the 2, 2′-azino-bis-3-ethylbenzthiazoline-6-sulphonic acid (ABTS) radical scavenging activity of the different plant fractions. The ferric reducing antioxidant power (FRAP) of the different solvent fractions was assessed according to the method (9).

The percentage yield after extraction with the various solvents was 15.9% for methanol, 11.0% for hexane, 6.3% for chloroform, 4.6% for ethyl acetate, 18.3% for butanol, and 46.4% for water (Table 1).

**Table 1:** Total phenolics and flavonoids of different fractions extract from *Aster glehni
*

***Fraction*** ^*^	***Total phenolics (mg GAE/g DW)***	***Total flavonoids (mg QE/g DW)***
Methanol	109.3±0.3	15.2±0.2
Hexane	48.5±0.5	14.7±0.3
Chloroform	52.5±0.4	5.4±0.5
Ethyl acetate	422.5±1.7	114.7±0.6
Butanol	158.5±0.7	28.6±0.1
Water	62.1±0.9	7.6±0.2

* Each sample concentration was 1.0mg/mL. Values are expressed as mean±standard deviation (n=3)

Phenol content was expressed in mg of gallic acid equivalent (GAE) per gram (mg GAE/g). The ethyl acetate extract had the highest phenol content of 422.5 ± 1.7 mg GAE/g, while the hexane extract had the lowest amount (48.5 ± 0.5 mg GAE/g). The butanolic and methanolic extracts resulted in 158.5 ± 0.7 mg GAE/g and 109.3 ± 0.3 mg GAE/g of phenol, respectively. The flavonoid content of the crude extracts was determined using quercetin as a standard and expressed as its equivalent (mg QE/g). The ethyl acetate fraction had higher flavonoid content (114.7 ± 0.6 mg QE/g) than the other solvent fractions. Butanol, methanol, and hexanol fractions had flavonoid contents of 28.6 ± 0.1 mg QE/g, 15.2 ± 0.2 mg QE/g, and 14.7 ± 0.3 mg QE/mg, respectively.

**Fig. 1:**
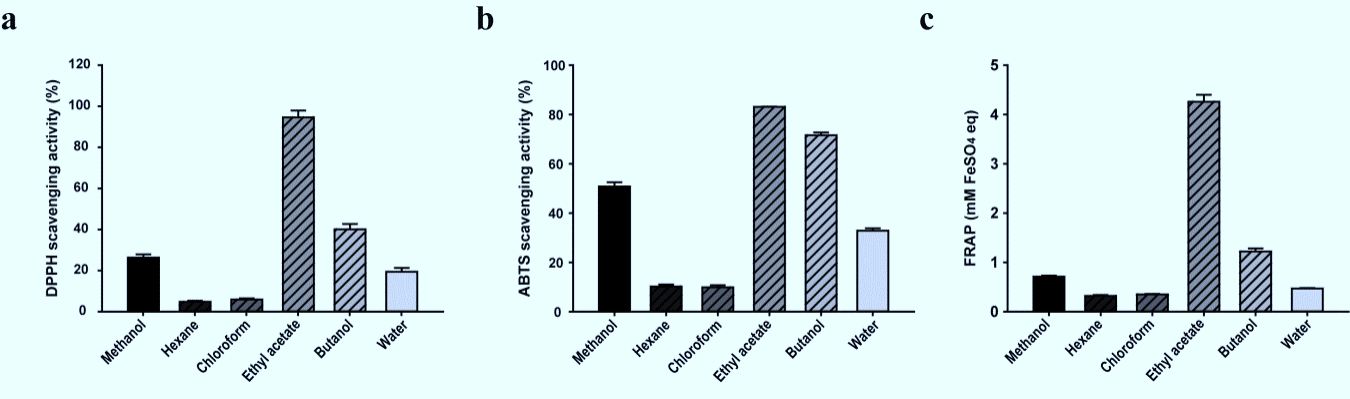
1, 1-diphenyl-2-picrylhydrazyl (DPPH) radical scavenging activity (a), 2, 2′-azino-bis-3-ethylbenzthiazoline-6-sulphonic acid (ABTS) radical scavenging activity (b), and ferric reducing activity (c) of the different solvent fractions of *Aster glehni*. Values are means ± standard deviation of three replications

The DPPH radical scavenging activity of the different solvent extracts was examined and compared to the activity of the known antioxidant butylated hydroxytoluene (BHT) at a concentration of 1.0 mg/mL. At 1.0 mg/mL, almost all of the solvent fractions exerted inhibitory activity on the DPPH radical, which ranged from 94.6% in the ethyl acetate fraction to 4.8% in the hexane extract. The scavenging activity of the extracts, in order, was: ethyl acetate > butanol > methanol > aqueous > chloroform > hexane (Fig. 1a). Similarly, all of the solvent extracts showed a wide range of ABTS radical scavenging activities at a concentration of 1.0 mg/mL. The ethyl acetate, butanol, and methanol extracts showed inhibitory activity against ABTS of over 50%. The order of extract activity was: ethyl acetate > butanol > methanol > aqueous > hexane > chloroform (Fig. 1b). The reducing power of the solvent extracts ranged from 4.26 mM FeSO_4_ equivalent/mg sample (ethyl acetate fraction) to 0.32 mM FeSO_4_ equivalent/mg sample (hexane fraction) at a sample concentration of 1.0 mg/mL. The reducing power activity of the solvent extracts was: ethyl acetate > butanol > methanol > aqueous > chloroform > hexane (Fig. 1c).

The results of this study showed that *A. glehni* contains large amounts of polyphenolic compounds and can exhibit considerable antioxidant activities in specific solvent fractions, including ethyl acetate and butanol fractions. Therefore, *A. glehni* may significantly reduce oxidative stress and has potential as a therapeutic agent for diseases related to oxidative stress.
